# SEOM-GEICO clinical guidelines on endometrial cancer (2021)

**DOI:** 10.1007/s12094-022-02799-7

**Published:** 2022-03-21

**Authors:** María Pilar Barretina-Ginesta, María Quindós, Jesús Damián Alarcón, Carmen Esteban, Lydia Gaba, César Gómez, José Alejandro Pérez Fidalgo, Ignacio Romero, Ana Santaballa, María Jesús Rubio-Pérez

**Affiliations:** 1 Medical Oncology Department, Institut Català d’Oncologia (ICO), Department of Medical Sciences, Girona Biomedical Research Institute (IDIBGI). Department of Medical Sciences, Medical School University of Girona (UdG), Girona, Spain; 2 Medical Oncology Department, Complexo Hospitalario Universitario de A Coruña. Biomedical Research Institute (INIBIC), A Coruña, Spain; 3 Medical Oncology Department, Hospital Universitari Son Espases, Fundació Institut d’Investigació Sanitària Illes Balears (IdISBa), Palma, Spain; 4 Medical Oncology Department, Complejo Hospitalario de Toledo, Toledo, Spain; 5 Medical Oncology Department, Hospital Clínic of Barcelona, Therapeutics in Solid Tumors, Translational Genomic and Targeted, Institut d’Investigacions Biomèdiques August Pi i Sunyer (IDIBAPS), Barcelona, Spain; 6 Department of Medical Oncology, Infanta Sofía and Henares Hospitals Foundation for Biomedical Research and Innovation (FIIB HUIS HHEN), Infanta Sofía University Hospital, Madrid, Spain; 7 Medical Oncology Department, Hospital Clínico Universitario of Valencia. Biomedical Research Institute INCLIVA. CIBERONC, Valencia, Spain; 8 Department of Medical Oncology, Fundación Instituto Valenciano de Oncología (IVO), Valencia, Spain; 9 Medical Oncology Department, Hospital Universitari i Politècnic La Fe, Valencia, Spain; 10 Medical Oncology Department, Hospital Universitario Reina Sofía. University of Córdoba, Córdoba, Spain

**Keywords:** Endometrial cancer, Guideline, Diagnosis, Treatment

## Abstract

Endometrial cancer (EC) is the second most common gynecological malignancy worldwide, the first in developed countries [Sung et al. in CA Cancer J Clin 71:209–249, 2021]. Although a majority is diagnosed at an early stage with a low risk of relapse, an important proportion of patients will relapse. Better knowledge of molecular abnormalities is crucial to identify high-risk groups in early stages as well as for recurrent or metastatic disease for whom adjuvant treatment must be personalized. The objective of this guide is to summarize the current evidence for the diagnosis, treatment, and follow-up of EC, and to provide evidence-based recommendations for clinical practice.

**Supplementary Information:**

The online version contains supplementary material available at 10.1007/s12094-022-02799-7.

## Introduction

Endometrial cancer (EC) is the most common gynecological cancer, following cervical cancer, in developed countries. Most patients are diagnosed at an early stage with a low risk of relapse. In the last 30 years, incidence has increased in a proportion of 1% per year, associated with higher mortality. Age at diagnosis and comorbidities (e.g. diabetes, hypertension and obesity) make treatment of EC challenging and might increase mortality. The high rate of cure in initial stages with an OS at 5 years around 80–85% have created the false belief that EC is a low-risk disease. Yet, advanced stages and some histologies are associated with poor prognosis.

## Methodology

This guideline is based on relevant published studies and with the consensus of ten EC treatment expert oncologists from GEICO (Spanish Group for Investigation in Ovarian Cancer) and SEOM (Spanish Society of Medical Oncology). The Infectious Diseases Society of America-US Public Health Service Grading System for Ranking Recommendations in Clinical Guidelines [2] has been used to assign levels of evidence and grades of recommendation.

## Diagnosis

The most frequent symptom of EC is abnormal uterine bleeding. In postmenopausal women or those with risk factors, metrorrhagia should always be investigated. Transvaginal ultrasound (TVUS) is usually performed given its availability and low cost. The cutoff level of 3 mm for exclusion of EC in women with postmenopausal bleeding is widely recommended [II, B], whereas no established consensus for premenopausal women [3] has been established. Histologic confirmation is always required. Blind endometrial biopsy is preferred although false negative results are frequent. In such cases, hysteroscopy with targeted biopsy could be recommended. In patients who cannot tolerate an office biopsy or for those with an unsuccessful office procedure, dilation and curettage, with or without hysteroscopy, is an option.

Preoperative imaging and histologic features help in tailoring the surgical approach to avoid unnecessary lymphadenectomy (LND) in low-risk patients [4]. TVUS can accurately evaluate myometrial invasion in most cases (80%) but it is less sensitive for cervical stromal invasion. Contrast-enhanced magnetic resonance imaging is the best method for detecting myometrial invasion or cervical involvement and it is highly recommended especially when conservative fertility preservation treatment is planned and in inoperable patients referred to radical radiation. At least an abdomino-pelvic computerized tomography scan (CTscan) must be performed to rule out lymph node (LN) or distant metastasis. Positron emission tomography/CTscan can also be employed. Thorax CTscan should also be performed as part of the initial assessment to exclude lung metastases in high-risk cases. The role of serum tumor markers is unclear [IV, B].

## Hereditary endometrial cancer

Around 5% of EC cases have an inherited mutation in Mismatch Repair (MMR) genes, namely MLH1, MSH2, MSH6, PMS2 or EPCAM [5]. The diagnosis of Lynch Syndrome is based on the detection of the germline mutation. Screening of Lynch Syndrome is currently recommended for all EC cases with no limitations regarding the age or the histology type [6] [IIA]. Screening of Lynch syndrome is based in the detection of MMR protein loss (MMR deficiency). When MMR-D is present, to rule out a non-hereditary cause, the MLH1 methylation or, more rarely, a BRAF mutation can be performed. If none of these are identified, a germline analysis of MMR genes must be undertaken. When carriers are identified, they must be advised of the specific lifetime risk of colorectal cancer ranging from 20% in PMS2 to 70% in MLH1 and other Lynch syndrome tumors. Moreover, this should prompt the direct mutation analysis of relatives to help identify carriers and to offer women prophylactic salpingo-ophorectomy and hysterectomy once childbearing is completed [IV, B].

Other less frequent EC hereditary syndromes can be caused by *PTEN* germline mutations in Cowden syndrome, (< 1%) [7], germline mutation in *BRCA1/2* (1%) with controversy regarding the association to serous histology [8] and germline TP53 mutations in Li-Fraumeni syndrome (< 1%).

## Screening

There are no high-quality data to support the efficacy of screening with imaging, tissue sampling, or cervical cytology for reducing EC mortality. Thus, in women with average or high-risk for endometrial cancer without abnormal bleeding, routine screening is not recommended [II, A]. This includes patients on tamoxifen, although there are no reliable data in women with extended therapy beyond five years.

Women with Lynch syndrome have a lifetime risk of endometrial cancer of 13–71%. Annual endometrial sampling, TVUS and CA125 beginning at age 30–35 or 5–10 years prior to the earliest age of first diagnosis of Lynch-associated cancer of any kind in the family is recommended [IV, B].

## Pathology and molecular biology of EC

Epithelial EC is divided into different histologic subtypes:

1. Endometrioid carcinoma.
2. Serous carcinoma.
3. Clear cell carcinoma.
4. Uterine carcinosarcoma.
5. Other: mucinous, neuroendocrine, undifferentiated, dedifferentiated carcinomas.

Endometrioid adenocarcinomas (EEC) are the most frequent subtype (≅ 80%). EEC is a heterogeneous subgroup that varies from indolent to very aggressive carcinomas. After tumor stage, the next most informative prognostic division in EEC is between high grade (grade 3) and low grade (grade 1–2). Despite the three-grade classification is widely used, a binary FIGO (International Federation of Gynecology and Obstetrics) grade is recommended: low grade (1–2) vs high grade (3) [9].

Serous carcinomas (SC) are the second most frequent endometrial carcinomas (< 10%). This subtype is aggressive and is frequently associated with deep myometrial involvement and lymphovascular invasion. More than 90% of SC are associated with *TP53* mutations.

Clear cell carcinomas (CCC) are a rare subgroup (1–6%) characterized by the clearing of tumor cell cytoplasm. Patients with CCC are more likely to present with a higher FIGO stage than EEC and have a poorer prognosis [10].

Uterine carcinosarcoma (UCS) is a highly aggressive tumor with a mixture of malignant epithelial and mesenchymal/sarcomatous components. UCS is very rare (≅ 1.5%). Next generation sequencing (NGS) analyses have revealed that UCS are serous-like tumors. The sarcoma dominance (presence of > 50% of sarcomatous element) is associated with worse prognosis [11].

Eventually EEC can coexist in the presence of SC or CCC, when one of these components are present in at least 5% it is classified as a mixed carcinoma.

Every subtype has been associated with different molecular alterations [12]. Table 1.

**Table 1:** Frequency of most common targeted alterations in endometrial cancer according to histological subtype

	EEC	SC	CCC	UCS
PTEN mutation	64–80% G1–3 52–82% G1/G2 62–90% G3	2–3%	0–21%	11–33%
PI3KCA mutation	22–59% G1–G3 38–54% G1/G2 45–59% G3	15–35%	24–36%	22–40%
PIK3R1 mutation	9–43% G1–G3 19–38% G1/G2 31–41% G3	5–8%	7–18%	6–20%
KRAS mutation	19–43% G1–G3 17–23% G1/G2 7–33% G3	2–6%	2–14%	10–17%
FGFR2 mutation	10–18% G1–G3 11–13% G1/G2 14–16% G3	8%	0%	0–2%
CTTNB1 mutation	19–37% G1–G3 24–28% G1/G2 19–40% G3	0–3%	0%	0–5%
MMR-d	34–35% G1–G3 34% G1/G2 44% G3	0–3%	11–14%	3–6%
ARID1A mutation	39–55% G1–G3 39–47% G1/G2 39–60% G3	7–11%	14–21%	10–24%
P53 mutation	5–14% G1–G3 6–10% G1/G2 21–35% G3	59–93%	28–46%	44–91%
ERBB2 amplification	1% G1–G3 3% G1/G2 4% G3	26–44%	11%	9%
POLE mutation	13–15% G1–G3 11% G1/G2 20% G3	0–2%	2–7%	3–4%

Modified from Urick and Bell [12]

## Molecular classification

In 2013, The Cancer Genome Atlas (TCGA) Research Network published an integrated genomic analysis of 373 EC. The analyses identified four prognostic categories of EC: *POLE* (ultra-mutated) (7%), microsatellite instability (MSI)/hypermutated (28%), copy number low/microsatellite stable (39%), and serous-like/copy number high (26%) [13].

POLE ultra-mutated is a subgroup characterized by somatic mutations in polymerase epsilon DNA polymerase (*POLE*) exonuclease domain which results in a high mutation rate. *POLE* is more frequently presented in low-grade and high-grade EEC. This subtype has an excellent prognosis and rarely recurs.

Microsatellite instability high (MSI-h) subtype is characterized by a deficiency in the MMR system that leads to an hypermutated status. Most MSI-h tumors are EEC.

The copy number (CN) low subgroup also called microsatellite stable, includes most of low-grade endometrioid tumors.

The CN high subgroup is frequently harbors *TP53* suppressor gene mutations and includes almost all SC and most of mixed type carcinomas and carcinosarcomas.

The complexity of the TCGA classification led to establishing more practical and feasible molecular classifications. The Leiden/TransPORTEC and the Vancouver/PROMISE studies generated two new classifications that are similar in molecular alterations. Although they are not identical to TCGA, these new classifications are preferred for clinical practice. They established four subgroups according to MSI status, *POLE* and *TP53* mutations. These four subgroups defined in thePORTEC study are:

1. POLE-mutant.
2. Microsatellite instable.
3. p53 abnormal.
4. No specific molecular profile (NSMP) [14] (Table 2).

**Table 2:**
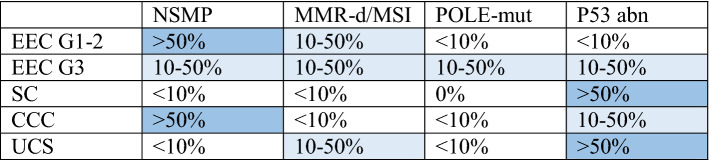
Relationship between histotype and molecular classification

Modified from Huvila J et al. [15]

## Staging and risk assessment

FIGO 2009 is the staging system currently recommended (Table 3). EC is surgically staged [16]. Risk groups have been designed based on clinicopathological factors associated at risk of recurrence to identify patients who may benefit from adjuvant therapy. Currently, well-defined clinicopathologic prognostic factors include: histological subtype, tumor differentiation grade, FIGO stage, age, depth of myometrial invasion and lymphovascular space invasion (LVSI) [17]. All these factors must be reported in the pathology report. LVSI should be categorized as (1) absent, (2) focal or (3) substantial. Focal LVSI is defined as the presence of a single focus and substantial LVSI as the presence of diffuse or multiple foci invasion or the identification of tumor cells in at least 5 lymphovascular spaces. Only substantial LVSI has been identified as prognostic for recurrence. Given its prognostic relevance, it is highly recommended to categorize tumors according to molecular classification [III,A], especially in the heterogenous high-grade EC subgroup that ranges from indolent POLEmut to highly aggressive p53 abnormal tumors. Molecular classification may be not required in low-risk EEC. According to the risk of relapse, EC can be subdivided into five risk categories integrating molecular markers (Table 4) [18].

**Table 3:** 2009 FIGO staging system for endometrial cancer

Stage I	Tumor confined to corpus uteri, including endocervical glandular involvement
IA	Tumor limited to the endometrium or invading less than half the myometrium
IB	Tumor invading one half or more of the myometrium
Stage II	Tumor invading the stromal connective tissue of the cervix but not extending beyond the uterus
Stage III	Tumor involving serosa, adnexa, vagina, or parametrium
IIIA	Tumor involving the serosa and/or adnexa (direct extension or metastasis)
IIIB	Vaginal involvement (direct extension or metastasis) or parametrial involvement
IIIC1	Regional lymph node metastasis to pelvic lymph nodes
IIIC2	Regional lymph node metastasis to para-aortic lymph nodes, with or without positive pelvic lymph nodes
Stage IV	Tumor invading bladder and/or bowel mucosa, and or distant metastasis
IVA	Tumor invading the bladder mucosa and/or bowel mucosa
IVB	Distant metastasis (includes metastasis to inguinal lymph nodes, intra-peritoneal disease, lung, liver, or bone)

**Table 4:** Prognostic risk groups—treatment recommendation

Risk group	Previous description	Molecular adapted	
Low	Stage I endometrioid G1–2, < 50% myometrial invasion, LVSI negative	Stage I–II POLEmut endometrial carcinoma,no residual disease Stage IA MMRd/NSMP endometrioid carcinoma G1-2, with no or focal LVSI	No adjuvant treatment
Intermediate	Stage I endometrioid, G1–2, ≥ 50% myometrial invasion, LVSI negative Stage I endometrioid G3, < 50% myometrial invasion and LVSI negative	Stage IB MMRd/NSMP endometrioid carcinoma G1-2, with no or focal LVSI Stage IA MMRd/NSMP endometrioid carcinoma G3, with no or focal LVSI Stage IA p53abn without myometrial invasion Stage IA non-endometrioid (serous, clear cell, undifferentiated carcinoma, carcinosarcoma, mixed) without myometrial invasion	BVT
High-intermediate	Stage I EEC with substancial LVSI regardless of grade and myometrial invasion Stage I EEC, G3, ≥ 50% myometrial invasion, regardless of LVSI Stage II	Stage I MMRd/NSMP endometrioid carcinoma, with LVSI Stage IB MMRd/NSMP endometrioid carcinoma G3 Stage II MMRd/NSMP endometrioid carcinoma	Surgical staging negative: VBT No surgical staging: PRT + VBT Consider CT for high grade or substantial LVSI
High	Stage III-IVA EEC optimally debulked Non-endometrioid EC (serous or clear cell or undifferentiated carcinoma, or carcinosarcoma)	Stage III–IVA MMRd/NSMP endometrioid carcinoma with no residual disease Stage I–IVA p53abn endometrial carcinoma with myometrial invasion, with no residual disease Stage I–IVA NSMP/MMRd serous, undifferentiated carcinoma, carcinosarcoma, with myometrial invasion, with no residual disease	EBRT concurrent or sequential with CT CT alone as alternative
Advanced metastatic	Stage III–IVA with residual disease or Stage IVB	Stage III–IVA with residual disease of any molecular type Stage IVB regardless molecular type	CT (RT can be considered depending on residual disease)

*p53abn* p53 abnormal, *POLEmut* polymerase-mutated, *LVSI* lymphovascular space invasion, *MMRd* mismatch repair deficient, *NSMP* non-specific molecular profile

For stage III–IVA POLEmut endometrial carcinoma and stage I–IVA MMRd or NSMP clear cell carcinoma with myometrial invasion, insufficient data are available to allocate these patients to a prognostic risk group in the molecular classification. Prospective registries are recommended

## Surgical treatment

### Early stages

All patients with newly diagnosed EC should be considered for surgery. Surgical staging is necessary for an accurate prognostic stratification and for adjuvant treatment decisions. Standard surgery in early stages is total hysterectomy with bilateral salpingo-oophorectomy without vaginal cuff resection. Peritoneal cytology, although considered a poor prognosis factor, is not mandatory for FIGO staging [19].

Minimally invasive surgery (laparoscopic or robotic) is the preferred surgical approach. Laparoscopic-assisted vaginal hysterectomy has been associated with lower peri and post-operative morbidity compared to laparotomy with similar oncologic outcomes [20]. Robotic surgery could be an alternative to laparoscopic approach, especially in those patients who have a high risk of conversion to laparotomy (e.g. obese patients). Additional routes (laparotomy or vaginal) can be individualized based on patient and tumor specific factors (e.g. uterine size, known adhesive disease or anesthesia limitations). Tumor rupture or morcellation should be avoided due to intra-peritoneal cells spillage risk.

LN evaluation provides prognostic information and could determine the adjuvant therapy. As the risk of LN involvement increases with tumor grade, high-risk histology, and depth of myometrial invasion, systematic pelvic and para-aortic LND in all patients is not recommended. Two randomized clinical trials demonstrated that systematic LND was not associated with overall survival (OS) or recurrence-free survival benefit in early-stage EC [21, 22]. In those patients considered for LN staging, uterine factors are used to assess the risk of retroperitoneal LN metastasis. If pelvic LN involvement is detected, para-aortic LN staging should be considered.

Sentinel node biopsy (SLND) has been introduced as an alternative to LND for LN staging, although there are no randomized clinical trials comparing outcomes between these two approaches. Multiple prospective cohort studies have demonstrated the feasibility of this technique with high sensitivity for detection of positive LN, in association with lower rates of post-operative morbidity (eg, lymphedema and cellulitis) [23]*.*

In SC, UCS or undifferentiated EC omentectomy should be included as a part of the staging procedure because of the high risk of omental metastases.

Recommendations:

- Standard surgical treatment in early stages EC is total hysterectomy and bilateral salpingo-oophorectomy without vaginal cuff resection with a minimally invasive surgery approach [I,A].
- In low-risk EC, systematic LND is not recommended [II, A]. In intermediate and high-risk group, LND is recommended to guide surgical staging and adjuvant therapy [II,C]. SNLB can be considered for staging purposes [III,A].
- Omentectomy should be performed in serous, carcinosarcoma, and undifferentiated endometrial carcinoma [IV,B].

## Advanced stages

In stage III-IV EC determination of whether cytoreduction is feasible is highly recommended. Surgical tumor debulking with complete macroscopic disease resection should be considered only in patients with good performance status and acceptable morbidity [III,B] [24]. Visible or palpable LN should be removed.

Palliative surgery could be considered in patients with good performance status and metastatic disease [IV,A].

## Fertility sparing therapy

Conservative management to preserve reproductive function and delay surgery until childbearing completion should only be performed in specialized centers. It should only be offered to patients with low-grade EEC without myometrial invasion [V,A] [25].

Initial work-up with pelvic and abdominal imaging and endometrial sampling is necessary to assess cancer grade and depth of myometrial invasion. Close follow-up and confirmation of lesion regression are also mandatory.

The most common approach is progestin therapy (medroxyprogesterone acetate; 400–600 mg/day or megestrol acetate; 160–320 mg/day or an intrauterine device containing levonorgestrel) [IV,B].

## Adyuvant treatment

### Radiotherapy

Pelvic radiation (PRT) after surgery in stage I EC provides locoregional control without improvement in OS or disease-free survival (DFS). A randomized trial comparing vaginal brachytherapy (VBT) and observation in women with stage IA, grade 1 and 2 EEC showed no OS beneft in VBT group. VBT was associated with an increase in genitourinary symptoms [26]. The results of PORTEC-2 trial, comparing VBT vs PRT in the high–intermediate-risk group, showed that there were no differences in pelvic or vaginal recurrences, DFS and distant metastasis, VBT being less toxic [27]. VBT in combination with PRT was compared to VBT alone in patients with intermediate risk [28]. Addition of PRT improved locoregional control without any impact on OS. Acute gastrointestinal and urinary toxicity was superior in the combination group. Postoperative RT has been considered standard in high-risk EC group, although a comparative study of adjuvant radiation versus no treatment in this group of patients has not been conducted.

### Chemotherapy

Results of two old prospective randomized trials comparing external beam RT (EBRT) to chemotherapy (CT) did not show differences in DFS an OS [29, 30]. CT reduced the risk of distant recurrences, but not that of local relapses. These observations provided the rationale for a combined CT/RT approach.

The pooled analysis of NSGO-EC-9501/EORTC-55991 and MaNGO ILIADE-III trials demonstrated that combined treatment (four cycles of platinum-based CT, given either before or after RT) improves DFS and showed a trend towards improved OS [31]. The limitation of these studies is that 25–40% of the patient population was stage III or incompletely surgically staged. The type of CT used and the number of cycles are other concerns that preclude generalization of these results.

In PORTEC-3 trial, EBRT was compared with chemoradiation (two cycles of cisplatin with EBRT, followed by four cycles of carboplatin and paclitaxel). The combined approach improved DFS and OS. The magnitude of benefit was greater in stage III and SC, but adverse events were more frequent with CT/RT [32]. Molecular analysis of PORTEC-3 trial participants suggested no benefit of CT for MMRd carcinomas [33]. GOG 258 trial compared CT (carboplatin and paclitaxel) vs CT-RT (two cycles of cisplatin with EBRT, followed by four cycles of carboplatin and paclitaxel) in stages III to IVA and stage I–II SC or CCC. There were no differences in DFS and OS [34], however the rates of locoregional relapses were higher with CT alone. In GOG 249, which included stage I EEC high–intermediate-risk group, stage I SC and CCC or stage II patients of any histology, CT (carboplatin-paclitaxel) plus VBT demonstrated similar DFS as EBRT, but more acute toxicity in chemotherapy arm [35].

### Adjuvant treatment. Recommendations (Table 4)

Incorporation of the molecular classification for adjuvant treatment decisions is encouraged, especially in high-grade tumors or high-risk disease where adjuvant chemotherapy is being considered. If molecular classification is not available, EC risk classification should be based on pathologic features.

Low-risk patients do not require adjuvant treatment [I,A].

VBT is recommended for intermediate-risk patients [I,A].

In the intermediate–high risk group, VBT is recommended in patients with surgical staging and node negative [III,B]. In patients with no surgical nodal staging, PRT and VBT is recommended [III,B]. Although adjuvant CT in intermediate high-risk group is not recommended, it can be considered in selected cases, especially for high grade and/or substantial LVSI [III, B].

In high-risk disease:

Adjuvant CT with EBRT (concurrent or sequential) is recommended [I,A]. Alternative option could be CT alone. [I,B].

p53abn identifies a high-risk group regardless of stage (except stage IA), histology and grade [IV,B].

*POLE*mut is associated with excellent prognosis and adjuvant therapy might be avoided in stage I-II disease [IV,B].

## Treatment of metastatic or recurrent disease

For pelvic isolated relapses or single metastatic sites, surgical resection, radiotherapy or ablative therapy should be considered [IV,A], as well as systemic therapy although its benefit is uncertain [IV,B].

In patients with recurrent unresectable or metastatic disease, CT and HT are therapeutic options. Enrollment in clinical trials is strongly recommended [V,B].

## Systemic treatment

Hormonal agents evaluated include progestogens alone or alternated with tamoxifen, tamoxifen alone, aromatase inhibitors and fulvestrant. Confirmation of hormone-receptor status by biopsy should be considered at the time of recurrence [IV,B]. The response rate (RR) in CT-naive patients is about 10–25% [36]. Hormonal therapy could be an appropriate therapeutic alternative for patients who are low-grade, hormone-receptor positive, without rapid progressive metastatic disease [37] [II,A]. The treatment of choice are progestogens (megestrol acetate 160 mg QD or medroxyprogesterone acetate 200 mg QD) or progestogens alternating with tamoxifen [III,A].

For more aggressive diseases, chemotherapy is the treatment of choice. Several combinations have been tested. GOG 209 showed equivalence of carboplatin-doxorrubicin-paclitaxel and carboplatin-paclitaxel with a PFS of 12–14 months and OS of 32 months, with a better toxicity profile for the latter [38]. Based on these results, the standard chemotherapy treatment for advanced or recurrent EC is the combination of carboplatin-paclitaxel [I, A]. For patients with late relapses (i.e. more than 6 months after last platinum), rechallenge with CT may be of beneficial [V,C].

## Immunotherapy

Several anti PD-1 and anti PD-L1 checkpoint inhibitors have shown activity in EC. Pembrolizumab showed activity in a phase II trial including patients with MMR-D tumors with 20% complete responses and 33% partial responses among EC patients [39, 40]. The phase II KEYNOTE-158 multicohort study evaluated the antitumor activity and safety of pembrolizumab in patients previously treated for advanced MSI-h/MMR-D non-colorectal cancer with 27 different histologies [41]. Among 49 patients with MSI-h/MMR-D EC, RR was 57.1%, and 16.3% of patients had a complete response. Median PFS was 25.7 months, and median OS was not reached.

The phase I GARNET trial evaluated the safety and activity of dostarlimab. The MSI-h cohort included 104 patients [42]. Approximately half of patients had received 2 or more prior lines of therapy. RR was 42.3%, 12.7% of patients had a complete response, and 29.6% had a partial response. With a median follow-up of 11.6 months, the estimated likelihood of maintaining a response was 96.4% at 6 months and 76.8% at 12 months in the MSI-h cohort.

The combination of Lenvatinib plus pembrolizumab showed promising activity in a phase II study in unselected patients with EC who had progressed to at least one previous line of treatment [43]. The phase III KEYNOTE-775 study evaluated the combination of lenvatinib plus pembrolizumab versus treatment of physician´s choice following platinum-based therapy in advanced EC [44]. 827 patients with advanced EC (unselected for MMR) were included, and about 85% of patients had MMR-proficient tumors. The combination of lenvantinib and pembrolizumab showed, regardless of MMR status, statistically significant improvements in OS (17.4 vs 12.0 months in MMR-proficient patients, and 18.3 vs 11.4 months in all-comers), PFS (6.5 vs 3.8 months in MMR-proficient patients, and 7.2 vs 3.8 months in all-comers). However, the combination of lenvatinib and pembrolizumab was associated with considerable toxicity; grade 3 treatment-related adverse events occurred in 88.9% of patients, and 33% of patients discontinued treatment because of treatment-related adverse events.

The combination of pembrolizumab and lenvatinib should be considered for second-line treatment of EC [I,A], particularly for MMR-proficient tumors, whereas Dostarlimab or pembrolizumab can be also considered for second-line therapy of MMR-D EC [II,B].

## Targeted therapies

Better knowledge of driver mutations across different EC subtypes has led to the development of multiple clinical trials with antiangiogenic agents, anti-HER2-targeted agents, PI3Kinase/mTOR, CDK4/6 and MEK inhibitors showing activity but without strong evidence to recommend its use. Inhibitors of other targets like PARP, WEE1, and the PI3K/AKT/mTOR pathway are subjects of thorough study.

## Follow up

Surveillance in EC is aimed at the early detection of recurrent disease. Most recurrences are diagnosed within 3 years of primary treatment. The most common site of recurrence is the pelvis, especially in the vaginal vault while distant relapses represent one-third of cases.

There is no evidence that any specific posttreatment surveillance strategy is associated with improved survival.

The TOTEM study [45] assessed the role of intensive (INT) vs minimalist follow-up (MIN). EC patients were included in two different cohorts: 1) low-risk group (FIGO IA G1-2) or 2) high-risk group (IA G3 or ≥ IB). The rate of relapse was 12.3%. No differences in OS were seen. According to TOTEM trial MIN strategy (clinical examination every 6/12 months for low-risk group and clinical examination and CTscan every 6/12 months for high-risk group) could be recommended for the follow-up of FIGO I-II EEC [I,B].

Surveillance consists mainly of monitoring symptoms and physical examination including: a speculum and pelvic examination every 3–6 months for 2 years, and every 6–12 months thereafter. Patients with low-risk endometrial cancer can be followed less frequently: 6–12 months for first 2 years, then yearly thereafter. Vaginal cytology is not routinely recommended as most vaginal recurrences are detected with clinical examination alone [I,A]. CA-125 may be used in surveillance for those patients who have an elevated CA-125 prior to treatment, advanced disease or serous endometrial cancer [46].

In high-risk non-endometrioid or FIGO III-IV tumors, imaging may be helpful, chest/abdominal/pelvic CT every 6 months during the first 3 years, and every 6 to 12 months for 2 additional years is recommended [IV,A]. Additional imaging considerations include whole body PET/CT in selected patients who may be candidates for surgery or locoregional therapy and/or pelvic MRI for patients who retain their uterus [47].

Following treatment, endometrial cancer patients should be counseled on the impact of obesity, lifestyle and nutrition [48] [IV,A].

## Supplementary Information

Below is the link to the electronic supplementary material.

Supplementary file1 (DOCX 18 KB)
