# Supplementary material for: SEOM-GEICO clinical guidelines on endometrial cancer (2021)
Source: Clin Transl Oncol. 2022 Mar 21;24(4):625–34. doi: 10.1007/s12094-022-02799-7 (PMC8986694; doi:10.1007/s12094-022-02799-7)
Supplement: Supplementary file 1 — Supplementary file1 (DOCX 18 KB) [file 12094_2022_2799_MOESM1_ESM.docx]

SEOM guidelines recommendations for the management of endometrial cancer

Diagnosis

- TVUS, with 3mm cutoff level for postmenopausal women, and endometrial biopsy is the standard approach [II,B]
- At least an abdomino-pelvic computerized tomography scan (CTscan) must be performed in order to rule out lymph node (LN) or distant metastasis. Positron emission tomography/CTscan can also be employed. Thorax CTscan should also be performed as part of the initial assessment to exclude lung metastases in high-risk cases. The role of serum tumor markers is unclear [IV, B].

Hereditary endometrial cancer:

- Screening for Lynch Syndrome is currently recommended for all EC cases with no limitations regarding the age or the histology type [IIA].
- Once Lynch Syndrome is diagnosed, direct mutation analysis of relatives to identify carriers and offer women prophylactic salpingo-ophorectomy and hysterectomy once childbearing is completed is recommended [IV, B].

Screening:

- In women with average or high-risk for endometrial cancer without abnormal bleeding, routine screening is not recommended [II, A].
- For women with Lynch Syndrome, annual endometrial sampling, TVUS and CA125 beginning at age 30 to 35 or 5 to 10 years prior to the earliest age of first diagnosis of Lynch-associated cancer of any kind in the family is recommended [IV, B].

Staging and risk assessement:

- EC is surgically staged. The staging is based on FIGO 2009 [IV, A]
- It is highly recommended to categorize tumors according to molecular classification [III,A]

Surgical treatment:

- Standard surgical treatment in early stages EC is total hysterectomy and bilateral salpingo-oophorectomy without vaginal cuff resection with a minimally invasive surgery approach [I,A].
- In low-risk EC, systematic LND is not recommended [II, A]. In intermediate and high-risk group, LND is recommended to guide surgical staging and adjuvant therapy [II,C]. SNLB can be considered for staging purposes [III,A].
- Omentectomy should be performed in serous, carcinosarcoma, and undifferentiated endometrial carcinoma [IV,B].
- Surgical tumour debulking with complete macroscopic disease resection should be considered only in patients with good performance status and acceptable morbidity [III,B].
- Palliative surgery could be considered in patients with good performance status and metastatic disease [IV,A].
- Fertility sparing surgery should only be offered to patients with low grade EEC without myometrial invasion [V,A].

Adjunvant treatment:

- Low-risk patients do not require adjuvant treatment [I,A]
- VBT is recommended for intermediate-risk patients [I,A]
- In the intermediate–high-intermediate risk group, VBT is recommended in patients with surgical staging and node negative [III,B]. In patients with no surgical nodal staging, PRT and VBT is recommended [III,B]. *Although adjuvant CT in intermediate high risk group is not recommended, it can be considered in selected cases, especially for high grade and/or substantial LVSI* [III, B].
- In high-risk disease:
  - Adjuvant CT with EBRT (concurrent or sequential) is recommended [I,A]. Alternative option could be CT alone. [I,B]
  - p53abn identifies a high risk-group regardless of stage (except stage IA), histology and grade [IV,B].
  - *POLE*mut is associated with excellent prognosis and adjuvant therapy might be avoided in stage I-II disease [IV,B].

Metastatic or recurrent disease:

- For pelvic isolated relapses or single metastatic sites, surgical resection, radiotherapy or ablative therapy should be considered [IV,A], as well as systemic therapy although its benefit is uncertain [IV,B].
- Hormonal therapy could be an appropiated therapeutic alternative for patients with low grade, hormone-receptor positive, without rapid progressive metastatic disease [II,A]. The treatment of choice are progestogens (megestrol acetate 160 mg QD or medroxyprogesterone acetate 200 mg QD) or progestogens alternating with tamoxifen [III,A].
- The standard chemotherapy treatment for advanced or recurrent EC is the combination of carboplatin-paclitaxel [I, A].
- For patients with late relapses (i.e. more than 6 months after last platinum), rechallenge with CT may be of benefit [V,C].
- The combination of pembrolizumab and lenvatinib should be considered for second-line treatment of EC [I,A], particularly for MMR-proficient tumors, whereas Dostarlimab or pembrolizumab can be also considered for second-line therapy of MMR-D EC [II,B].

Follow up:

- According to TOTEM trial MIN strategy (clinical examination every 6/12 months for low-risk group and clinical examination and CTscan every 6/12 months for high-risk group) could be recommended for the follow up of FIGO I-II EEC [I,B].
- Vaginal cytology is not routinely recommended as most vaginal recurrences are detected with clinical examination alone [I,A].
- In high risk non-endometrioid or FIGO III-IV tumors, imaging may be helpful, chest/abdominal/pelvic CT every 6 months during the first 3 years, and every 6 to 12 months for 2 additional years is recommended [IV,A]
- Following treatment, endometrial cancer patients should be counseled on the impact of obesity, lifestyle and nutrition [IV,A]
